# The effects of teaching strategies on learning to think critically in primary and secondary schools: an overview of systematic reviews

**DOI:** 10.12688/f1000research.158087.2

**Published:** 2025-04-16

**Authors:** Andrew D. Oxman, Allen Nsangi, Laura Martínez García, Margaret Kaseje, Laura Samsó Jofra, Daniel Semakula, Heather Munthe-Kaas, Sarah E. Rosenbaum

**Affiliations:** 1 Centre for Epidemic Interventions Research (CEIR), Norwegian Institute of Public Health, Oslo, Oslo, 0213, Norway; 2 College of Health Sciences, Makerere University, Kampala, Central Region, Uganda; 3 CIBER of Epidemiology and Public Health, Barcelona, Spain; 4 Iberoamerican Cochrane Centre, Sant Pau Biomedical Research Institute, Hospital de la Santa Creu i Sant Pau, Barcelona, Catalonia, Spain; 5 Tropical Institute of Community Health and Development in Africa, Kisumu, Kenya; 6 Epidemiology and Public Health Department, Hospital de la Santa Creu i Sant Pau, Barcelona, Catalonia, Spain

**Keywords:** Teaching strategies, Critical thinking, Health, Systematic reviews

## Abstract

**Background:**

We conducted an overview of systematic reviews about the effects of teaching strategies that can be used to teach primary and secondary school students to think critically. Our objective was to inform decisions about what teaching strategies to use in resources that we developed to teach critical thinking about health in secondary schools.

**Methods:**

We mapped characteristics of systematic reviews of teaching strategies and summarised findings from the most relevant reviews to teaching students to think critically about health. We included reviews that assessed the effects of teaching strategies that could potentially be used in primary or secondary schools to teach students to think critically, had a Methods section with explicit selection criteria, reported at least one outcome measure of the ability to undertake one of four basic types of cognitive tasks (memory, procedural, comprehension, or opinion), and were published after 1999.

**Results:**

We included 326 systematic reviews. The reviews evaluated a wide range of teaching strategies for a variety of purposes. Important limitations of the reviews included not considering adverse effects (99% of the reviews), not assessing the risk of bias for included studies (93% of the reviews), and not assessing the credibility of subgroup effects (100% of the reviews). We summarised the findings for 37 teaching strategies that we considered most relevant. The certainty of the evidence of the effects varied from very low to moderate. We used 12 of the strategies in resources that we developed to teach secondary students to think critically about health.

**Conclusions:**

A tremendous amount of work has gone into evaluating the effects of a wide range of teaching strategies. The results of this research can inform decisions about how to teach critical thinking and future research. However, well-designed, up-to-date systematic reviews are still needed for many teaching strategies.

## Introduction

### Critical thinking about health

We designed the *Be Smart about your Health* resources for secondary school teachers and their students (in the 8 ^th^ or 9 ^th^ year of school, age 13-15) in Kenya, Rwanda, and Uganda. ^1^ The aim of these resources is to help students cope with the abundance of information and misinformation about how to care for their health. Critical thinking is important for making informed choices and it is a core competency in the curricula for all three countries. ^2– 4^ However, it is often not taught in the classroom, partly due to a lack of carefully developed educational resources, and a lack of information about effective teaching strategies. The *Be Smart about your Health* resources were developed to help fill this gap. These resources focus on critical thinking about health actions – things that people do to care for their health or the health of others. Critical thinking about health actions is especially important because:

• Good health is essential for daily life and depends on informed health choices, which depend on critical thinking about health actions.
• Health is important to everyone, which makes it a good starting point for learning critical thinking skills that students can transfer to other subjects, such as the environment. ^5^

The *Be Smart about your Health* resources focus on nine key concepts that people need to understand and apply when deciding what to believe about the effects of health actions and what to do. ^6^^,^^7^ We undertook this overview of systematic reviews of the effects of teaching strategies to inform the design those specific resources. However, the results from this review can also be applied more broadly to inform the development of other critical thinking learning resources. Given the growing use of large language model and artificial intelligence that young people are exposed to, coupled with a deluge of mis/disinformation, critical thinking skills are increasingly important to ensure that young people have the necessary skills to identify reliable evidence and make informed choices. In response, many educational systems have begun to recognize the importance of critical thinking by including these skills in curriculum reforms, national and international standardized tests (e.g., Programme for International Student Assessment; PISA), and by incorporating problem-based learning approaches in the classroom. ^8^ However, the implementation of teaching strategies to facilitate and support the development of critical thinking skills varies widely across subjects, schools and countries. ^9^^,^^10^

### How we have conceptualised critical thinking

Learning to think critically is widely held to be an aim of education. ^11^ However, there is not agreement on the definition of “critical thinking”, or which frameworks (conceptual structures intended to serve as a support or guide) best support critical thinking. ^12– 16^ Ennis has defined critical thinking as “reasoned, reflective thinking focused on deciding what to believe or do”, ^17^ and we use that definition in this overview. ^18^

Thinking evolved to help us choose what to do to achieve our goals after taking account of estimates of the likely effects of our actions. ^19^ A fundamental goal of critical thinking is to improve decision making by increasing the likelihood that we will believe and act on those claims that are more likely to help us achieve our goals. ^20^

### What we mean by “teaching strategies”

Definitions of teaching (instructional, or pedagogical) strategies (techniques, methods, or approaches) vary. Some authors distinguish between strategies, techniques, methods, and approaches. However, there is overlap in how these terms are used. Our focus is on “different ways of helping students to learn - that is, different ways of helping them to achieve the learning outcomes that [teachers] have decided are important”. ^21^ We refer to these as “teaching strategies”.

There are several lists of teaching strategies, organised in different ways. ^21– 27^ Beck surveyed 25 teacher education textbooks and was unable to find two similar lists of teaching strategies. ^22^ Pomerance and colleagues reviewed 48 textbooks for elementary and secondary teacher training and found that none of the textbooks accurately described six fundamental instructional strategies. ^27^ At most, only two of the six were covered in any textbook, and when textbooks did mention the strategies (allowing for a broad range of terminology and descriptions), the discussion could be as brief as 1-2 sentences.

### Why we have taken a broad focus in this overview

Although our specific interest is in primary and secondary school students and critical thinking outcomes, we have not limited this overview to that population or those outcome measures. There are four reasons for this. First, there are not many reviews that focus specifically on critical thinking. ^28– 39^ To the extent that those reviews do consider the effects of specific strategies, they tend to be broad categories and comparisons of strategies tend to be made indirectly (in meta-regression analyses). For example, Abrami and colleagues explored differences in the effect of three types of instruction (dialogue; authentic or anchored instruction; and mentoring, coaching, or tutoring) across 341 comparisons with different populations, outcome measures, and study designs. ^28^ Thus, an overview that only included critical thinking as an outcome would be limited.

Second, although some learning outcomes may be of little relevance to learning to think critically, it is difficult to specify a priori which outcomes are completely irrelevant and which might provide useful information despite not directly measuring critical thinking. For example, on the one hand it can be argued that outcome measures that only require retention of knowledge are irrelevant to critical thinking. On the other hand, it is important that students have knowledge of key concepts (principles for critical thinking) ^7, 40^ and that they retain that knowledge. Other outcome measures, such as reading comprehension or understanding of science texts, are dependent on a range of factors in addition to critical thinking.

Third, many reviews are not limited to primary or secondary school interventions and may or may not explore differences in effects across different students. Although some teaching strategies might be expected to have different effects for different types of students, it is uncertain whether this is the case. Starting out with an overly narrow focus in terms of the students could result in an overview that is far less informative than it might otherwise be.

Fourth, we were unsure how many potentially useful systematic reviews of teaching strategies there were and what the characteristics of those reviews were.

For these reasons, we conducted an overview to characterise the range of systematic reviews of teaching strategies that can potentially inform the design of resources to help primary and secondary school students learn to think critically. The overview may be useful to researchers, teachers, policymakers, and others with an interest in other learning outcomes and students. It enabled us to make an informed decision about which reviews were most useful for our specific interests. ^1^

## Objectives

Our primary objectives were to provide an overview of what is known from systematic reviews about the effects of teaching strategies that can be used to teach primary and secondary school students to think critically and inform the design of educational resources (the *Be Smart about your Health* resources) to teach lower secondary school students in East Africa to think critically about health claims and choices.

Secondary objectives were to:

• Map characteristics of systematic reviews of teaching strategies
• Identify needs and priorities to assess teaching strategies based on the findings of the included systematic reviews
• Identify needs and priorities to assess systematic reviews of the effects of teaching strategies
• Inform the development of a framework for types of teaching strategies

## Methods

The protocol for this overview was published in Zenodo in December 2019. ^41^

### Criteria for considering systematic reviews for inclusion

We included systematic reviews that:

• assess the effects of teaching strategies (different ways of helping students to learn) that can potentially be used in primary or secondary schools to teach students to think critically,
• have a Methods section with explicit selection criteria,
• report at least one outcome measure of the ability to undertake one of four basic types of cognitive tasks (memory, procedural, comprehension, or opinion), ^42^ and
• were published after 1999.

We excluded reviews of teaching strategies that were restricted to:

• professional students (e.g., medical or nursing students) other than teacher training
• special education (teaching children and youth with disabilities)
• creative or physical skills such as artistic, cooking, musical, or physical skills

Doyle defined the four basic types of cognitive tasks noted above as follows ^42^:

1. memory tasks in which students are expected to recognize or reproduce information previously encountered (e.g., memorize a list of spelling words or lines from a poem);
2. procedural or routine tasks in which students are expected to apply a standardized and predictable formula or algorithm to generate answers (e.g., solve a set of subtraction problems);
3. comprehension or understanding tasks in which students are expected to (a) recognize transformed or paraphrased versions of information previously encountered, (b) apply procedures to new problems or decide from among several procedures those which are applicable to a particular problem (e.g., solve “word problems” in mathematics), or (c) draw inferences from previously encountered information or procedures (e.g., make predictions about a chemical reaction or devise an alternative formula for squaring a number);
4. opinion tasks in which students are expected to state a preference for something (e.g., select a favourite short story).

These tasks roughly correspond with Bloom’s taxonomy, which has six main categories of intellectual abilities and skills: knowledge, comprehension, application, analysis, synthesis, and evaluation. ^43^ Bloom’s taxonomy is well known and has clear definitions, but it difficult to make clear distinctions between the higher-order categories. ^14^ For the purposes of this overview, we considered any task that requires judgement (‘evaluation’ in Bloom’s taxonomy) as ‘opinion tasks’ including judgements about what to believe and what to do.

### Search methods for identification of systematic reviews

We created an initial list of potentially relevant teaching strategies ( Box 1) by reviewing several lists. ^21– 26, 44^ We started with Beck’s taxonomy, ^22^ which we adapted and reorganised, considering other teaching strategies and ways of categorising these. We continued to develop this list of terms iteratively, based on the literature that we retrieved and input from educational researchers and teachers.

Box 1. **List of teaching strategies.**

**Didactic strategies** (instruction in which information is presented directly from the teacher to the student, in which the teacher selects the topic, controls instructional stimuli, obligates a response from the student, evaluates responses, and provides reinforcement for correct responses and feedback for incorrect ones)

Direct instruction, lectures, textbooks, picture books, audio-visual aids, podcasts, multimedia instruction, demonstration, modelling, mini lessons, reading, graphic presentations, combined graphic and verbal presentations, narrative text, comics, humour, scaffolding, pre-teaching vocabulary, link abstract concepts with concrete representations

**Questioning techniques** (methods used for constructing and presenting questions in order to promote effective discussions and learning or to elicit information)

Socratic method, open ended questions, closed questions, interviewing, prompting, probing, redirecting, wait time, clickers, pose probing questions, oral or written reports, cloze, “assess to boost retention”, quizzes, ask and answer deep questions

**Discussion strategies**

Classroom discussion, small group discussion, buzz sessions, brainstorming, round table, debate, structured controversy, magic circle, fishbowl dialogue, four sides/corners strategy, reflective discussion, flipped classroom

**Role playing**

Read aloud, readers’ theatre, dramatic play, storytelling, mock trial, simulation, learning games, public speaking, and speech writing

**Problem-based learning**

Enquiry-based learning, exploration-based learning, student research, research projects, learning through experimentation, science fairs, science Olympics, using case studies to teach, laboratory teaching methods, field trips, discovery learning, analytic memo, concept attainment, concept formation, concept maps, graphic organizer, knowledge map, cognitive organiser, mind mapping, structured overview, “repeatedly alternating problems with their solutions provided and problems that students must solve”

**Repetition and progression**

Distributed practice, space learning over time, spaced learning, pacing, learning targets, learning progression, competency-based learning, sequential approach, explicit teaching, interdisciplinary teaching

**Assessment techniques**

Feedback, classroom assessment techniques, formative assessment, background knowledge probe, the one-minute paper, traffic light cards, muddiest point, what’s the principle, problem recognition task, student generated test questions, classroom opinion polls, directed paraphrasing, pro and con grid, student goals ranking, course-related interest and skills checklist, self-diagnostic learning logs, misconception/preconception check, empty outlines, invented dialogues, diagnostic teaching, precision teaching

**Collaborative learning** (a situation in which two or more people learn or attempt to learn something together)

Dyads, partners, cross/multi-age groups, ability and interest groups, heterogeneous groups, homogeneous groups, cooperative learning, heads together strategy, numbered heads together strategy, jigsaw teaching technique, team learning, peer teaching, peer partner learning, reciprocal teaching, readers’ workshop, reading buddies, think-ink-pair-share learning strategy, think-pair-share learning strategy, heterogeneous grouping, homogeneous grouping, multiple intelligences activities

**Individual learning**

Individualized instruction, learner-controlled instruction, self-paced learning, independent study, programmed learning, contract learning, mastery learning, tutorial instruction, learning centres, menus, course packets, teaching tailored to students’ learning styles, Dalton plan, writing, writing to inform, paraphrasing, pause and reflect, journal writing, homework, practice, anchor activities

**E-learning** (using electronic devices, applications, or processes to acquire or transfer knowledge, attitudes, or skills through study, instruction, or experience)

Online learning, web-based learning, web-based instruction, Web Quest, computer-based training, mobile learning, virtual classrooms, webinars, interactive e-lessons, online discussions, electronic simulations, audio/video recording

**In-service teacher training**

Microteaching, powerful pedagogical strategies, team teaching, scaffolding, peer teaching, teachers’ guides, or any of the other teaching strategies listed above

Starting with the terms in Box 1, we developed search strategies for Education Research Complete (EBSCO) and for Education Resources Information Center (ERIC) (Ovid). ^73^ The searches were conducted October 13, 2019 (EBSCO) and October 29, 2019 (ERIC). We updated the searches March 15, 2022 (ERIC) and March 22, 2022 (EBSCO).

### Selection of systematic reviews

Two authors independently screened the titles and abstracts identified in October 2019 to identify systematic reviews that met our inclusion criteria. Disagreements were resolved by discussion, involving a third author if needed. We pilot tested the selection criteria on a sample of 100 records as training, before screening the search results. We retrieved the full text of articles that appeared to meet the selection criteria and two authors independently assessed each article for inclusion. Cochrane Response ^45^ screened the updated search and selected reviews for inclusion in the same way.

### Data collection

For each systematic review included in the overview two review authors independently collected the following data:

• Year of the last search
• Number of included studies
• Countries where included studies were done
• School subjects
• Education level of participants
• Age of participants
• Teaching strategies that were evaluated and their definitions
• Outcome measures included in the review
• Consideration of adverse effects
• Included study designs (randomized trials, non-randomized studies, mixed)
• Assessment of the risk of bias for effect estimates
• Limitations of estimates for effect modifiers considered by the review authors

Cochrane Response collected the data for included reviews identified by the updated search in the same way.

### Data synthesis

We mapped characteristics of the included systematic reviews. We used a framework thematic synthesis approach to categorise the teaching strategies. ^46, 47^ This entailed four stages: familiarisation, coding, charting and interpretation of the data. We started with the framework shown in Box 1. The definitions and boundaries of each category of strategies were discussed among the review authors, and the framework was revised in line with categories that emerged from the data.

We assessed the relevance of each included review for teaching critical thinking in primary and secondary schools. These judgements were discussed by the review team and a consensus was reached on the systematic reviews that were most relevant to the design of the *Be Smart about your Health* resources. For each teaching strategy that we considered relevant, one review author prepared a summary based on the included systematic reviews. The other authors reviewed and edited those summaries. Each summary included an explanation of the strategy, when and why it should be used, a bottom line, and a judgement of the certainty of the evidence for the bottom-line using Grading of Recommendations Assessment, Development and Evaluation (GRADE) criteria. ^48, 49^ We did not do this for the reviews identified by the updated search, which was conducted after the *Be Smart about your Health* resources had been designed and pilot tested.

### Ethics statement

Ethical approval was not required for this work.

## Results

**Figure 1.**
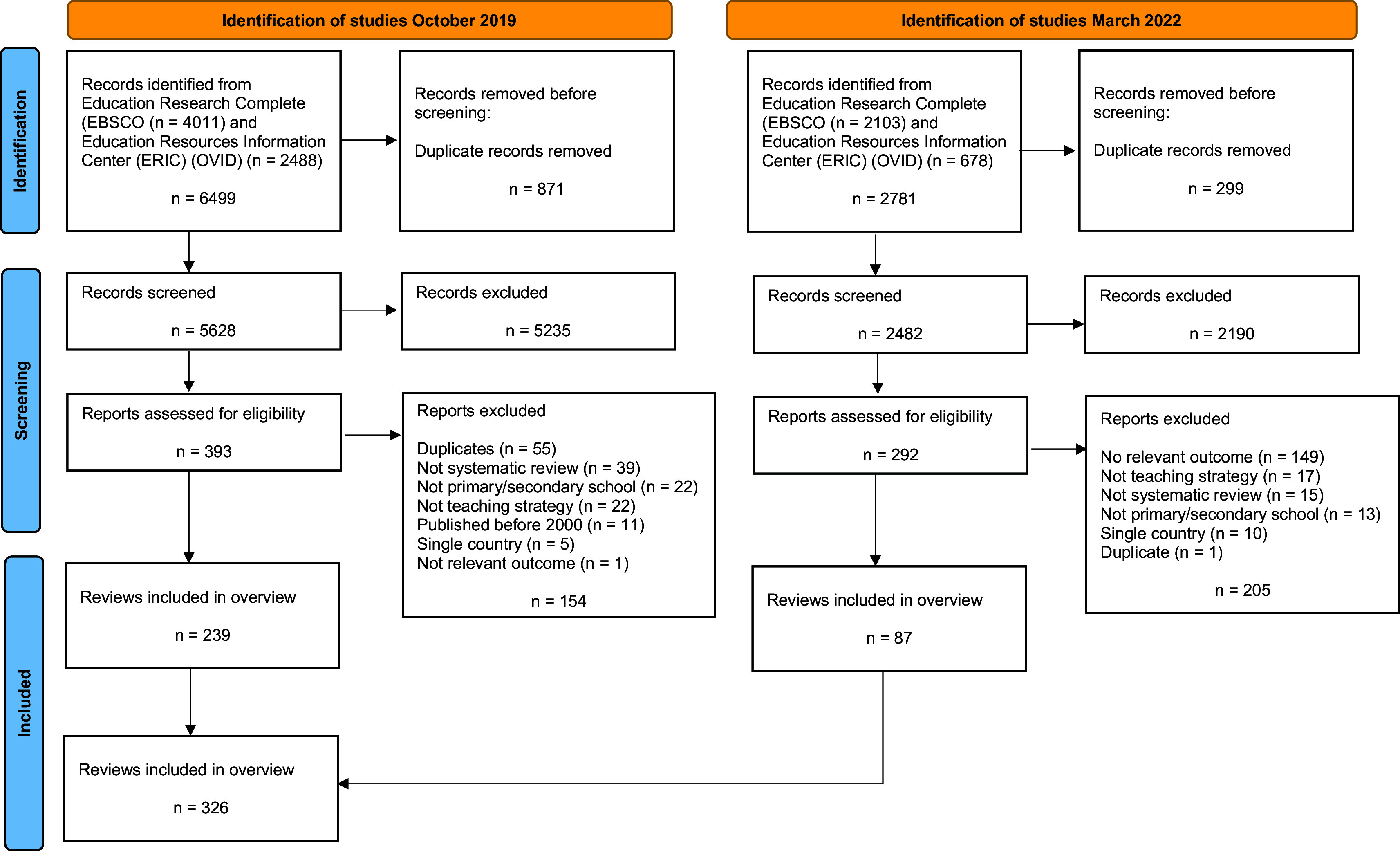
PRISMA flow diagram.

We identified 9280 records - 6499 records in the search conducted in October 2019 and 2781 records in the updated search conducted in March 2022 ( Figure 1). After removing duplicates, we screened a total of 8110 records and assessed 685 full-text reports for eligibility. We included a total of 326 systematic reviews of teaching strategies that reported at least one quantitative cognitive outcome measure applicable to primary or secondary schools. ^73^

**Figure 2.**
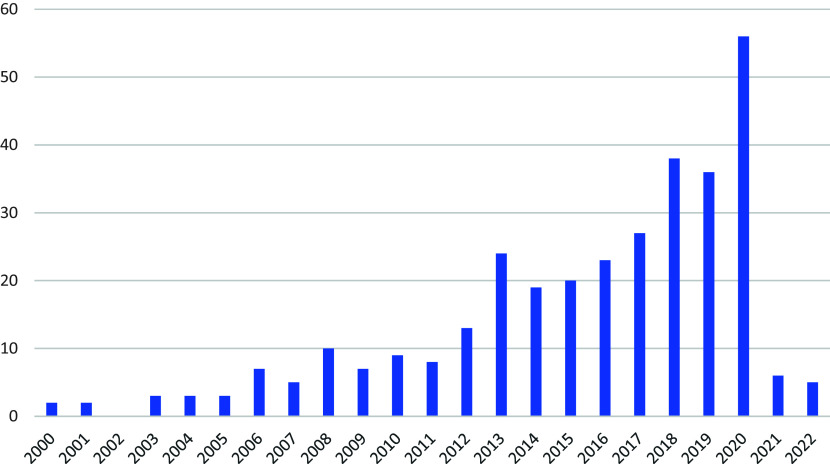
Number of systematic reviews published each year.

The number of systematic reviews of teaching strategies published each year increased from two in 2000 to 56 in 2020 ( Figure 2). Just over 40% of the reviews (132 reviews; 40.5%) were published in seven journals: Review of Educational Research (40; 12.3%), Educational Research Review (25; 7.7%), Educational Psychology Review (17; 5.2%), Computers & Education (15; 4.6%), Journal of Educational Psychology (15; 4.6%), Journal of Computer Assisted Learning (10; 3.1%), and Journal of Educational Computing Research (10; 3.1%).

Among the 226 reviews (69.3%) that reported the year of the last search, the difference between the year of publication and the year of the last search was two or more years for 151 reviews (64.5%). The year of the last search was not reported for 100 (30.7%) of the included reviews.

The reviews included between 5 and 1105 studies (median 34). The studies were conducted in over 60 different countries. Most studies were conducted in high-income countries. Studies from 16 countries were included in 10 or more reviews ( Table 1). Studies from the USA were included in at least 110 reviews (32.8%). However, 204 reviews (62.6%) did not report the countries in which included studies were conducted and other reviews only partially reported the countries in which included studies were conducted.

**Table 1.** Countries in which included studies were conducted.

Country	Studies included in reviews	
	n	%
USA	110	33.7%
Turkey	41	12.6%
Canada	40	12.3%
Taiwan	39	12.0%
UK	39	12.0%
Australia	31	9.5%
Netherlands	27	8.3%
Germany	24	7.4%
China	18	5.5%
Israel	17	5.2%
Spain	17	5.2%
Singapore	12	3.7%
Cyprus	10	3.1%
Hong Kong	10	3.1%
Malaysia	10	3.1%
Norway	10	3.1%

The education level of the included participants varied widely within and across reviews ( Table 2). Over 100 of the included reviews (104; 31.9%) included studies with participants ranging from preschool or primary school through university. Only 11.7% (38) of the reviews focused specifically on primary, middle, or secondary school. The reviews that included multiple education levels frequently did not explore education level as an effect modifier. Thirty-nine reviews (12.0%) did not report the education level of participants in the included studies or use educational level as a criterion for including studies in the review. Most of the included reviews (262; 80.4%) did not report the ages of participants in included studies or the ages corresponding to the included education levels.

**Table 2.** Education levels of participants in the included studies.

Education level	Systematic reviews	
	n	%
Preschool	1	0.3%
Preschool – primary school	5	1.5%
Preschool – middle school	1	0.3%
Preschool – secondary school	7	2.1%
Preschool – university	11	3.4%
Primary school	26	8.0%
Primary school – middle school	9	2.8%
Primary school – secondary school	62	19.0%
Primary school – university	93	28.5%
Middle school	3	0.9%
Middle school – secondary school	9	2.8%
Middle school – university	8	2.5%
Secondary school	9	2.8%
Secondary school – university	13	4.0%
University	30	9.2%
Not reported	39	12.0%
**Total**	**326**	**100%**

Many of the reviews (112; 34.4%) included studies in multiple school subjects, often without exploring the subject as a potential effect modifier ( Table 3). Nearly one third (100; 30.7%) of the reviews focused on math and science topics. Many of the reviews (92; 28.2%) did not report the subjects that were the focus of the included studies.

**Table 3.** School subjects that were the focus of studies included in the reviews.

School subjects	Systematic reviews	
	n	%
Multiple subjects	113	34.7%
Multiple science, math & science, or STEM subjects *	57	17.5%
Mathematics subjects	38	11.7%
Single science subjects	5	1.5%
Language or literacy	21	6.4%
Not reported	92	28.2%
**Total**	**326**	**100%**

* STEM includes science, technology, engineering, and mathematics.

### Teaching strategies

The included reviews addressed a wide range of teaching strategies, often using different terms to describe similar strategies, and using different definitions. ^73^ We grouped the teaching strategies into 11 broad categories ( Table 4). Altogether, 110 reviews (33.7%) addressed the use of information and communication technology in education (“E-learning”). Half (55) of those reviews fit in a different category than E-learning category. For example, we included digital games in the category “games and role play”.

**Table 4.** Categories of teaching strategies.

Category	Systematic reviews	
	n	%
**Didactic strategies** Instruction in which information is presented directly from the teacher or authors of instructional material to the student, in which the teacher or authors selects the topic and controls what is presented	27	8.3%
**Questioning and prompts** Methods used by teachers or authors of instructional material to present questions to students, elicit information, and promote effective discussions, reflection, and learning	8	2.5%
**Assessment and feedback** Evaluating students’ performance or progress and giving information to students with the aim of helping them to improve their performance and learn	13	4.0%
**Individual learning** Activities that individual students can do on in a classroom or at home, and instruction that is tailored to the needs, skills, and interests of individual students	73	22.4%
**Collaborative learning** Two or more students work together on a shared learning goal. Students are encouraged to ask questions, give explanations, exchange arguments, and formulate new ideas and solutions	30	9.2%
**Games and role play** Games are interactive, based on a set of rules and constraints, and directed toward a clear goal that is often set by a challenge. In addition, games constantly provide feedback either as a score or as changes in the game world to enable players to monitor their progress toward the goal. Role play allows students to immediately apply content as they are put in the role of a decision maker	27	8.3%
**Problem-based and inquiry learning** In problem-based learning, students work collaboratively to clarify and define a problem, propose solutions based on what they already know, and identify learning gaps associated with the problem. By engaging in self-directed learning, students work on filling those gaps, and conclude the process of learning by sharing their newly acquired knowledge and finalizing and presenting their solution. In inquiry-based instruction students learn by engaging in the thinking processes and activities of scientists, including asking scientifically oriented questions, collecting and analysing evidence, developing explanations of scientific phenomena, and communicating those explanations	41	12.6%
**E-learning ** Strategies using information and communication technology that did not fit into any of the above categories	55	16.9%
**Teacher training** Any type of continuing education effort or in-service training for educators	4	1.2%
**Systematic reviews of multiple teaching strategies** Systematic reviews that assessed the effects of several different categories of teaching strategies	38	11.7%
**Marginally relevant systematic reviews** Systematic reviews that met our inclusion criteria but address topics that are marginally relevant to informing the design of resources to help primary and secondary school students learn to think critically	10	3.1%
**Total**	**326**	**100%**

Twenty-two reviews addressed the use of games or gamification, most of which focused on digital games, and we found 16 reviews of flipped classrooms ( Table 5). On the other hand, we found only four reviews of teacher training that reported student outcomes, and only eight reviews of the effects of different approaches to questioning and prompting students.

**Table 5.** Teaching strategies evaluated by the included systematic reviews.

**Didactic strategies (n 27)**
Animation (3), Conversational style instructional text (1), Direct instruction (1), Digital media (1), E-books (1), Emotional design (1), Graphic presentations (1), Including the history and philosophy of science in science teaching (1), Integrated pictures and words (1), Multi-media instruction (2), Pedagogical agents (4), Seductive details (1), Shared storybook reading (1), Signalling (2), Teaching with PowerPoint (1), Technology-enhanced stories (1), User-paced segments for multimedia presentations (1), Using representations for mathematics (1), Verbal redundancy (1), Visual displays (1)
**Questioning and prompts (n 8)**
Asking high-level questions (1), Case comparisons (1), Cues (1), Explanation prompts (3), Response cards (1), Explanation prompts (3), Similarities and differences (2)
**Assessment and feedback (n 13)**
Computer-based assessment and feedback (1), Concept mapping for formative assessment (1), Digital badges (1), Feedback in computer assisted language learning (1), Formative assessment (3), Peer assessment (2), Practice tests (3), Self- and peer-grading (1)
**Individual learning (n 73)**
Adaptation to students’ needs (differentiation) (4), Adaptivity in digital games (1), Brain-based learning (1), Combining learning with physical activity (1), Computer-based personalised instruction (1), Computer-based scaffolding (4), Cover, copy, compare (1), Differentiated literature instruction (1), Digital flipped classroom (1), Digital metacognitive instruction (1), Digital scaffolding (1), Digital story telling (2), Digital tutoring (4), Example-based learning (1), Experiential learning (2), Flipped classroom (16), Grammar instruction (2), Guided notetaking (2), Homework (1), Homework and practice (1), In + out of class activities (1), Learner control with educational technology (1), Learning strategy instruction (1), Non-computerised working memory interventions (1), Notetaking (1), Notetaking and reviewing (1), Output tasks for vocabulary learning (1), Parent tutoring (1), Parental involvement (1), Parental involvement in homework (1), Programmed instruction (1), Reflective thinking (1), Self-regulation training (2), Student-centred learning and computer-aided instruction (1), Student-centred education (1), Teaching argument writing (1), Text structure instruction (1), Tutoring for literacy (1), Working memory training (2), Writing to read (1), Writing to learn (2)
**Collaborative learning (n 30)**
Collaborative problem solving (1), Computer-supported collaborative learning (2), Concept cartoons (1), Constructive and interactive activities (1), Cooperative learning (5), Grouping within class (1), Mobile supported collaborative learning (1), Peer feedback (1), Peer instruction (1), Peer tutoring (5), Peer-assisted learning (1), Peer-led learning (1), Peer-response (1), Small group discussion (1), Small group learning (2), Teacher guidance during collaborative learning (1), Team-based learning (2), Technology-supported student interaction (1)
**Games and role play (n 27)**
Competition in digital game-based learning (1), Creative drama (2), Digital games (11), Digital games and simulations (1), Drama-based learning (1), Game-based learning (4), Gamification (6), Learning games (1), Mobile games (1)
**Problem-based and inquiry learning (n 41)**
Active learning (1), Computer-based inquiry (1), Computer-based scaffolding (2), Concept mapping (2), Constructivist technology-intensive learning (1), Context-based learning (2), Creative thinking activities (1), Digital manipulatives (2), Discovery-based instruction (1), Failure-based learning (1), Incubation periods for problem solving (1), Inference instruction (1), Inquiry-based instruction (3), Manipulatives (2), Mobile inquiry-based learning (1), Modelling-based instruction (2), Philosophy for children (1), Problem posing (2), Problem solving (1), Problem-based learning (6), Project-based learning (2), Research projects (1), Scaffolding for problem solving (1), Schema instruction (1), Student-question-based inquiry (1), Word solving strategies (1)
**E-learning (n 55)**
Augmented reality (5), Blended learning (4), Computer programming (1), Computer simulations (1), Computer-assisted instruction (7), Digital tools (4), Distance education (4), Educational technology (8), Exploratory computerised environments (1), Laptop programs (1), Mobile learning (9), Online and blended learning (1), Online discussion (1), Online education (1), Robotics (2), Small group or individual learning using computers (1), Tablets (1), Touch devices (1), Virtual reality (2), Wearable technologies (1)
**Teacher training (n 4)**
Instructional development (1), Professional development (2), Teacher coaching (1)
**Systematic reviews of multiple teaching strategies (n 38)**
Interventions targeting working memory (1), Classroom instruction (1), Classroom management (1), Cooperative, competitive, and individualistic learning (1), Creative pedagogies (1), Critical thinking in e-learning (1), Critical thinking instruction (1), Early grade literacy interventions (1), Education interventions in Sub-Saharan Africa (1), Educational materials (1), Secondary school science programs (1), Explicit teaching of critical thinking to English language learners (1), Fraction interventions for struggling primary school (1), Improving learning in primary schools in developing countries (1), Innovative science and mathematics teaching (1), Instruction targeted at children who are at risk for language and reading comprehension difficulties (1), Instructional interventions for critical thinking (1), Interventions for elementary and middle school students with low socioeconomic status (1), Interventions targeted at students with or at risk of academic difficulties (1), Language comprehension interventions (1), Learning environment (1), Literacy interventions in low- and middle-income countries (1), Media literacy interventions (1), Middle and secondary school reading programs (1), Middle and secondary school mathematics programs (1), Primary school mathematics programs (1), Primary school reading programs (1), Primary school science programs (1), Professional development interventions for mathematics (1), Reading interventions for secondary school (1), Reading strategy interventions (1), Strategies for facilitating critical thinking in asynchronous online discussions (1), Strategies for teaching creativity (1), Strategies for teaching critical thinking (1), Teaching methods for secondary algebra (1), Teaching methods in chemistry (1), Teaching strategies for algebra (1), Vocabulary instructional strategies (1)
**Marginally relevant systematic reviews (n 10)**
A commercial cognitive-training program (1), A computer-based literacy program (1), Extensive reading for vocabulary (1), Balancing reading and writing in literacy programs (1), Family literacy interventions (1), Use of children's literature in English first language classrooms (1), Writing instruction (4),

### Outcome measures

The included reviews reported a wide range of measures of academic achievement (measured using standardized tests or grades) or learning (measured using researcher- or teacher-created tests). Twenty-six reviews (8.0%) reported a measure of transfer. Forty-six reviews (14.1%) reported a measure of higher order thinking, including critical thinking (11 reviews; 3.4%), problem solving (16; 4.9%), metacognition (8; 2.5%), and argumentation (5; 1.5%).

Three reviews mentioned adverse effects. One noted parenthetically in the authors’ conclusions that “We found … (and no significant adverse effects).” It did not refer to adverse effects anywhere else in the review. The other two reviews mentioned potential adverse effects (of parental involvement with homework, and of home environments). Two reviews mentioned practical disadvantages (student unfamiliarity and instructor start-up cost; and taking time away from instruction and students’ responses). Two reviews considered costs. Thirty-three reviews (10.1%) considered negative effects on the learning outcomes that were reported. The rest of the reviews (286; 87.7%) did not consider adverse effects, disadvantages, or costs.

### Study designs, risk of bias, and limitations

The study designs included in the reviews often were not clear ( Table 6). Reviews frequently included “Experimental” and “quasi-experimental” studies without providing clear definitions. In some reviews, “experimental” clearly referred to studies using random allocation, but that was not always clear. “Quasi-experimental” could refer to a variety of different study designs. Similarly, when “quantitative” or “comparative” studies were included, the designs of the included studies often were not described.

**Table 6.** Included study designs.

Study designs	n	%
“Experimental" and "quasi-experimental” *	157	48.2%
“Experimental” *	59	18.1%
Quantitative (comparative) ^†^	31	9.5%
“Quasi-experimental” *	18	5.5%
Quantitative and qualitative ^†^	21	6.4%
Case studies	4	1.2%
Not clearly reported	36	11.0%
**Total**	**326**	**100%**

* “Experimental” and “quasi-experimental” were not well defined. Experimental studies sometimes clearly referred to randomized studies. Quasi-experimental studies may include a variety of study designs.

^†^ “Quantitative” or “comparative” studies may include a variety of study designs and may or may not include randomized trials.

Twenty-four (7.4%) of the reviews assessed the risk of bias for included studies using explicit criteria. Twenty (6.1%) assessed “study quality” using explicit criteria, some of which are not directly related to the risk of bias, and four reviews (1.2%) did not clearly report the criteria that were used. Fifty-three reviews (20.2%) did not assess the risk of bias but included random allocation (53) or study design (13) as a potential effect moderator. The other 212 (65%) of the reviews did not address the risk of bias.

Most reviews (235; 72.1%) assessed potential effect moderators using univariate meta-regressions (173 reviews; 53.1%), multivariate meta-regression (25; 7.7%), or network meta-analysis (1 review). Thirty-six reviews (11.0%) reported effects for subgroups of included studies without a test of interaction (statistical significance of differences between subgroups). Of the 235 reviews that assessed potential effect modifiers, 39 (16.2%) addressed limitations of those analyses due to, for example, a lack of data (17 reviews), general limitations of moderator analyses (10 reviews), or potential confounding (7 reviews). None of the included reviews systematically assessed the credibility of reported subgroup effects.

### Findings of the most relevant systematic reviews

We prepared plain language summaries for 37 teaching strategies that we judged to be most relevant to teaching critical thinking in primary and secondary schools, and one for teacher training. ^73^ These summaries informed the choice of teaching strategies that we used in *Be Smart about your Health.* We included teaching strategies that could potentially be used to teach critical thinking, even though we did not find a systematic review that directly considered effects on critical thinking. We also included strategies that are relevant for learning new vocabulary (such as important new terms that are introduced in the resources) and for explaining and helping students to understand the key concepts that are taught in the resources.

For each summary we included when the review authors last searched for evidence. We also included a judgement about the certainty of the evidence, based on limitations of the included studies (our assessment based on information provided in the reviews), imprecision of the effect estimates, and inconsistency of results. We judged the certainty of the evidence to be moderate for 16 teaching strategies, low to moderate for one, low for 11, and very low for 10 ( Table 7). We did not judge the certainty of the evidence to by high for any of the teaching strategies.

**Table 7.** Certainty of the evidence and size of the effects for the most relevant systematic reviews.

Certainty of the evidence			Size of the effect	
Level of evidence	Definition	Term used in the bottom lines	Effect size	Term used in the bottom lines
Moderate	This research provides a good indication of the average effect. The actual effect is likely to be close to what was found, but there is a possibility that it will be substantially different.	Probably	< 0.1	Little or no effect
			0.1 – 0.29	Small effect
Low	This research provides some indication of the average effect, but the actual effect may be substantially different from that effect.	May	0.3 – 0.39	Small to moderate effect
			0.4 – 0.59	Moderate effect
Very low	The effect is very uncertain.	Uncertain	0.6 – 0.69	Moderate to large effect
			> 0.7	Large effect

**Figure 3.**
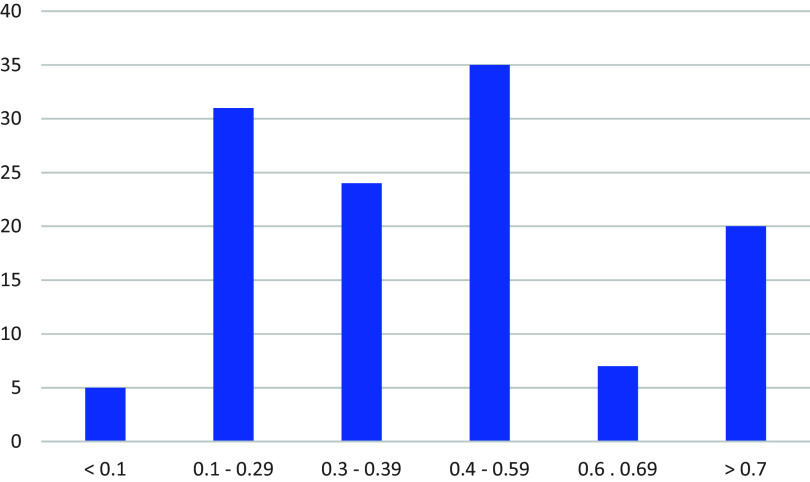
Average effect sizes from meta-analyses included in summaries of the most relevant teaching strategies vary greatly.

We categorised the size of the effects from little or no effect to a large effect, as shown in Table 7. The 122 average effect sizes that we included in the summaries ranged from 0.01 to 1.68, median 0.42 ( Figure 3). We wrote short descriptions and “bottom-line statements” for the teaching strategies that we considered most relevant. The bottom-line statements are shown in Table 8. The bottom-line statements communicate the certainty of the evidence and the size of the effects (when these were available). We constructed them by adapting strategies for writing informative sentences to communicate evidence from systematic reviews. ^50^

**Table 8.** Bottom-line statements for the most relevant teaching strategies *.

Strategy	Bottom line
**Didactic strategies** Didactic strategies are instruction in which information is presented directly from the teacher or authors of instructional material to the student, in which the teacher or authors selects the topic and controls what is presented.	
• **Signalling** Signalling is the use of a variety of methods to attract the learners’ attention and highlight important information (e.g., underlining) to improve learning outcomes. Text signalling includes organisation highlighting, picture referencing, colour, and intonation. Graphic signalling includes pointing gestures, colour, labelling, flashing, spotlight, and graphic organisers.	Signalling probably improves retention and transfer. Text signalling may be more effective than graphical signalling.
• **Integrated pictures and words** Integrated pictures and words or “integrated designs” are instructional designs that integrate relevant texts and images, in contrast to spatially distant designs that do not.	Integrated pictures and text are probably more effective for learning than spatially distant pictures and text.
• **Verbal redundancy** The term redundancy has been frequently used by researchers to describe the presentation of the same information through different presentational modes and sense modalities. Verbal redundancy is the concurrent presentation of text and verbatim speech.	Presenting written key terms together with spoken presentations may be more effective than spoken-only presentations or verbatim, spoken-written presentations.
• **Pedagogical agents** Pedagogical agents are characters that facilitate instruction.	Pedagogical agents, agent gesturing, and embodying affect in agents probably improve learning outcomes.
**Questioning and prompts** Questioning and prompts are methods used by teachers or authors of instructional material to present questions to students, elicit information, and promote effective discussions, reflection, and learning.	
• **Cues, questions, and advance organizers** Student learning is enhanced when teachers understand and capitalize on students’ prior knowledge and preconceptions about a topic. The strategy of using cues, questions, and advance organizers guides students from the known to the unknown by activating and, as appropriate, re-creating a cognitive framework of familiar concepts in which to incorporate new information. Advance organizers are introduced before a lesson to draw attention to important points, identify relationships within the material, and relate material to students’ prior knowledge.	Advance organizers are probably effective and, in general, it appears that strategies that help students activate existing knowledge and prepare a cognitive framework for new information increases their ability to assimilate new knowledge. However, the effectiveness of different types of cues and questions is uncertain.
• **Asking high-level questions** High-level questions address the top cognitive processes in Bloom’s taxonomy: i.e., they are questions that require analysis, synthesis, or evaluation.	Asking high-level questions and instructing students to ask high level questions may facilitate critical thinking and asking inappropriate questions may hinder critical thinking.
• **Explanation prompts** Self-explanation is a process by which students generate explanations for themselves to make sense of new information. Explanation prompts promote self-explanation.	Prompting students to generate explanations probably has a small to moderate effect on learning. Asking students to define or elaborate the meaning of concepts may have a large effect.
• **Response cards** Response cards are cards, signs, or items that are simultaneously held up by all students in the class to display their responses to questions or problems presented by the teacher to increase active student response.	Compared to hand raising, response cards probably increase participation, decrease off-task behaviours, and increase learning.
• **Similarities and differences** Using similarities and differences is a teaching strategy in which students are told to identify similarities and differences, compare, classify, or identify relationships or patterns among things or ideas.	Using similarities and differences probably improves learning. The size of the effect may be large when compared to traditional instruction and small when compared to interactive instruction.
**Assessment and feedback** Assessment is the process of evaluating students’ performance or progress. Feedback is information given to students with the aim of helping them to improve their performance and learn.	
• **Setting objectives and providing feedback** Setting objectives is the process of establishing a standard to guide learning. Closely aligned with setting clear goals for learning is providing feedback to learners on how they are doing in relation to the achievement of the goals.	Setting objectives and providing feedback probably improves learning, but the size of the effect may vary from small to large.
• **Reinforcing effort and providing recognition** One strategy to increase motivation is to reinforce effort. The theory behind this strategy is that the reinforcement should support a student’s effort rather than only recognizing ability. This allows a chance for all students to receive recognition, because all students can put forth effort.	The effects of reinforcing effort and providing recognition are uncertain.
• **Computer-based assessment and feedback** Feedback is information regarding aspects of a learner’s performance or understanding provided, for example, by a teacher or peer. Feedback can help students identify and correct errors and misconceptions, develop more effective and efficient problem-solving strategies, and improve their self-regulation. To do so, it must be processed mindfully.	Computer-based, elaborated feedback may improve critical thinking. Immediate feedback may improve lower order thinking, such as understanding terms.
• **Digital badges** A digital badge is a representation of an accomplishment, interest or affiliation that is visual, available online. It similar to awarding recognition via physical status icons, such as ribbons, medals, and trophies.	Digital (and physical) badges may improve learner engagement and motivation. However, they can also have a negative impact on engagement.
• **Practice tests** Students take a practice test on studied material before taking a final test on the same material.	Practice tests are probably more effective than restudying for both retention of information and transfer of knowledge or skills.
• **Self-grading and peer-grading ** Self-grading and peer-grading involves students making judgments about their own and others’ academic performance. They evaluate the extent to which performance criteria and standards have been met and provide criterion-referenced feedback, that is, grading, to themselves or others.	Self- and peer-grading probably improve learning.
**Individual learning** We have included here strategies that individual students can do on in a classroom or at home, as well as individualise instruction, which is tailored to the needs, skills, and interests of each student.	
• **Note taking and summarising** Note taking is the process of capturing key ideas and concepts. Summarising is the process of identifying essential information and restating it in a condensed form.	Note taking and summarising may improve learning and instructing students how to take notes and summarise may be beneficial, but the size of the effects is uncertain and may depend on the type of note taking and the instructional strategy that is used.
• **Non-linguistic representations** Non-linguistic representations are strategies that teachers can use to encourage students to create, store, and manipulate visual or non-linguistic representations either in their minds or with concrete tools and displays.	Teaching students to use non-linguistic representations is probably effective, but the size of the effect is uncertain.
• **Homework and practice** Homework (tasks assigned to students by teachers that are meant to be completed outside of school hours) can serve a variety of purposes, including opportunities to practice or review materials, introducing new materials, encouraging the transfer of skills to new situations, and integrating separately learned skills.	Homework may have a small effect on academic achievement. The benefits of homework are likely to depend on the nature of the homework and students’ circumstances.
• **Computer-based scaffolding for reading comprehension** Scaffolds are tools, strategies, and guides to support students in regulating their learning.	The effectiveness of various types of computer-based scaffolding for reading comprehension is uncertain.
• **Computer-based personalised instruction** Computer-based personalised instruction includes automated, adaptive guidance and technology programs. Automated, adaptive guidance is broadly defined as any guidance that occurs within computer-based instruction with two components: computer scoring of students’ work and computer delivery of a response to students’ work based on the automated scores.	Computer-based personalised instruction is probably effective, but the size of the effect varies.
• **Adaptation to students’ needs (differentiation)** Adaptation to students’ needs or differentiation is a combination of careful progress monitoring and adapting instruction in response. Feedback and progress monitoring is where teachers or students receive detailed information about students’ development, often with the objective of customising instruction to individual students’ needs.	Adaptive computerised instruction probably is effective; feedback and progress monitoring may be effective; homogeneous grouping probably harms low-ability students.
**Collaborative learning** In collaborative learning, two or more students work together on a shared learning goal. Students are encouraged to ask questions, give explanations, exchange arguments, and formulate new ideas and solutions. We have included concept cartoons here, which can be used as a stimulus for collaborative learning, although they can also be used in teacher-led classroom discussion.	
• **Constructive and interactive activities** Constructive activities require learners to create something that goes beyond the information given in the learning environment. Interactive activities engage learners in a dialogue in which they build upon the other people’s constructive contribution.	Constructive and interactive activities are probably effective for teaching scientific reasoning and scientific argumentation, but the size of the effect varies.
• **Cooperative learning** Cooperative learning is a group-based instructional strategy in which students work together under a particular set of conditions. These conditions are established to assuage the negative aspects of group behaviour while maintaining the benefits.	Cooperative learning probably improves learning.
• **Small group discussion** Small group discussion is a learner-centred, active-learning strategy. The aim is to stimulate learners’ interest in what they are studying by providing them with autonomy over the learning activity. It moves away from lessons dominated by the teacher talking.	Small-group discussion may improve students’ understanding of evidence and transfer of the knowledge and skills that are learned to new situations.
• **Teacher guidance during collaborative learning** Teacher guidance during collaborative learning refers to teachers making instructional decisions - monitoring the problems students encounter and intervening when necessary.	Teacher guidance during collaborative learning may have an important impact on the effectiveness of collaborative learning.
• **Computer-supported collaborative learning** Computer-supported collaborative learning is based on three main elements: collaboration, the use of computers, and the use of learning tools (e.g. communication, discussion, multimedia, or visual representation tools), or supporting strategies (e.g. teacher facilitation, peer feedback or assessment, or instruction and guidance).	Computer-supported collaborative learning probably improves learning.
• **Concept cartoons** Concept cartoons are visual tools in which cartoon characters declare different views (using short texts) about an event from daily life. They are used as the starting point for discussion and to encourage students to reveal and explain their views.	Using concept cartoons to teach scientific (critical) thinking probably is at least as effective as other teaching strategies and may be more effective for short-term learning goals.
**Games and role play** Games are interactive, based on a set of rules and constraints, and directed toward a clear goal that is often set by a challenge. In addition, games constantly provide feedback either as a score or as changes in the game world to enable players to monitor their progress toward the goal. Role play allows students to immediately apply content as they are put in the role of a decision maker.	
• **Digital games** Digital games, also referred to as serious games or game-based learning, are interactive, based on a set of rules and constraints, and directed toward a clear goal that is often set by a challenge. In addition, games constantly provide feedback either as a score or as changes in the game world to enable players to monitor their progress toward the goal.	Digital games probably improve learning, but the size of the effect varies.
• **Role play** Creative drama is the animation of a purpose or a thought based on the experiences of a group or group members by making use of techniques such as improvisation and role-playing.	Creative drama or role playing probably improves learning and skills, including higher thinking skills.
**Problem-based and inquiry learning** In problem-based learning, students work collaboratively to clarify and define a problem, propose solutions based on what they already know, and identify learning gaps associated with the problem. By engaging in self-directed learning, students work on filling those gaps, and conclude the process of learning by sharing their newly acquired knowledge and finalizing and presenting their solution. In inquiry-based instruction students learn by engaging in the thinking processes and activities of scientists, including asking scientifically oriented questions, collecting and analysing evidence, developing explanations of scientific phenomena, and communicating those explanations.	
• **Active learning** Active learning engages students in the process of learning through activities or discussion in class, as opposed to passively listening to an expert. It emphasizes higher order thinking and often involves group work.	Active learning is probably more effective than passively listening to a lecture, especially for higher-level cognitive skills, such as critical thinking.
• **Concept mapping** A concept map is a node-link diagram in which each node represents a concept, and each link identifies the relationship between the two concepts it connects.	Concept maps probably improve learning.
• **Applying knowledge to new problems** “Generating and testing hypotheses” is a technique that requires students to apply previous or developing knowledge to novel situations (problem solving).	Activities that involve applying knowledge to new problems probably improve learning compared to traditional instructional activities.
• **Inquiry-based instruction** In inquiry-based instruction students learn by engaging in the thinking processes and activities of scientists, including asking scientifically oriented questions, collecting and analysing evidence, developing explanations of scientific phenomena, and communicating those explanations.	Inquiry-based instruction probably improves learning and guidance probably improves the effectiveness of inquiry-based instruction.
• **Student question-based inquiry** Student-question-based inquiry in science education is the kind of inquiry that begins from the students’ questions.	The effects of student-question-based inquiry are uncertain.
• **Problem-based learning** In problem-based learning, students work collaboratively to clarify and define a problem, propose solutions based on what they already know, and identify learning gaps associated with the problem. By engaging in self-directed learning, students work on filling those gaps, and conclude the process of learning by sharing their newly acquired knowledge and finalizing and presenting their solution.	It is uncertain whether problem-based learning is more effective than traditional, lecture and discussion-based, teacher-controlled teaching strategies in increasing content knowledge, problem-solving ability, or critical thinking skills.
• **Scaffolding for inquiry-based learning** Scaffolds are tools, strategies, and guides to support learning by students. Computer-based scaffolding in inquiry-based or problem-based learning assists students as they generate solutions to complex and ill-structured problems, goals, or tasks, helping students enhance knowledge and higher order thinking skills.	Computer-based scaffolding for inquiry-based or problem-based learning is probably effective.
**Teacher training** Teacher training or professional development is any type of continuing education effort or in-service training for educators.	
Teacher training is often associated with one-time in-service lecture-based conferences, courses, or workshops taking place in a central location. However, teacher training can occur onsite or online and can involve many of the other teaching strategies reviewed above, mentoring, professional networks, or teacher coaching.	Although in-service teacher training is likely important for implementing change in schools, the effects of different approaches to teacher training are uncertain.

* References can be found in Oxman (2024). ^73^

Although many of the reviews had important limitations, they provided a valuable starting point for selecting and implementing teaching strategies that we incorporated in our educational resources and that teachers potentially can use to teach critical thinking. Some teaching strategies require more training and experience of teachers than others. We included information about the need for teacher training or experience, whenever this was addressed in a review.

## Discussion

Hundreds of systematic reviews of teaching strategies have been produced over the past 20 years. They provide a valuable basis for informing decisions about the use of these strategies. However, these reviews have some important limitations, including:

• Inconsistent terminology and definitions
• Unnecessary duplication of efforts
• Failure to consider adverse effects
• Inadequate assessments of the risk of bias
• Inadequate assessments of the credibility of subgroup effects (effect moderators)

We developed and revised a framework for categorising teaching strategies that met our needs. It is uncertain whether that framework will prove useful to others. Some of the reviews included in our overview could be classified in more than one way. Nonetheless, we found the framework helpful for organising the reviews and the teaching strategies and making sense of what is known. It would be helpful for users of this research if researchers studying the effects of teaching strategies could reach a consensus on a taxonomy. Similarly, it would be helpful if agreement could be reached on the terminology that is used to characterize outcome measures. Coordination by an organisation such as the Campbell Collaboration might help to reduce unnecessary duplication of efforts and make it easier to update the reviews. It might also help reduce the risk of bias in systematic reviews of teaching strategies. ^51^

Only three of the systematic reviews included in this overview even mentioned adverse effects. Researchers commonly fail to consider potential adverse effects of educational and public health interventions. ^52– 55^ An adverse effect on an intended outcome is sometimes found, but other types of adverse effects are rarely considered, including misunderstanding, misapplication of learning, distrust, cognitive dissonance, stress, inequities, conflict, and wasted time or resources.

Many of the systematic reviews did not assess the risk of bias for studies included in the reviews using explicit criteria. This made it difficult to judge the certainty of the evidence, i.e., confidence in the reported effect estimates. ^56– 58^ In addition, none of the reviews systematically assessed the credibility of reported subgroup (moderator) effects. ^59– 61^ The credibility of most subgroup claims in randomized trials, even strong claims, is usually low. ^62, 63^

### Strategies for teaching critical thinking in primary and secondary schools

Although we have included effect sizes in our summaries of strategies that we considered most relevant to teaching critical thinking in primary and secondary schools, the average effect sizes summarised in Figure 3, as well as those reported in other overviews such as Hattie’s, ^64^ are difficult to interpret for several reasons in addition to those noted above, including:

• Variable quality of the included meta-analyses
• Variable quality of the studies included in the meta-analyses ^65^
• Variable outcome measures
• Variation in what each strategy was compared to
• Variable statistical methods
• General challenges to interpreting effect sizes ^66^

For these reasons, the reported effect sizes should not be used to compare the effectiveness of different teaching strategies and firm conclusions cannot be drawn about the magnitude of effects for most of the teaching strategies.

Nonetheless, the findings of the systematic reviews that we considered most relevant for teaching critical thinking in primary and secondary schools helped to inform the design of the *Be Smart about your Health* resources. Because we developed the resources in East Africa, many of the teaching strategies that we considered potentially relevant in other settings were not practical in low-resource settings, particularly ones that depend on the availability of digital technology. We initially pilot tested resources that used different teaching strategies for each lesson. However, this was inefficient, since the strategies were new to both teachers and students, and this distracted from the learning goals of the lessons. Therefore, we selected a small number of strategies that we used consistently across lessons and only varied the main lesson strategy a few times ( Table 9). ^1^

**Table 9.** Teaching strategies used in the *Be Smart about your Health* resources.

Strategies used across lessons	Strategies used in individual lessons
• Guided notetaking • Small-group work • Response cards • Homework – collecting claims and choices about health actions • Standard lesson structure • Setting objectives and providing feedback • Multimedia design	• Concept mapping • Concept cartoons • Inquiry-based instruction • Quizzes • Role play

The *Be Smart about your Health* intervention is being evaluated in randomized trials and process evaluations in Kenya, Rwanda, and Uganda. ^67– 72^

Some of the teaching strategies included in this review may be more effective for some subjects than others, however this is outside the scope of this review, but could benefit from further investigation.

### Limitations

Limitations of this overview and our findings include:

• The searches were limited to two databases (Education Research Complete (EBSCO) and Education Resources Information Center (ERIC).
• Coding the included reviews was challenging and there were frequent disagreements.
• We did not systematically assess the risk of bias for included reviews.
• The assessments of the certainty of the evidence for reported effects were based on information provided in the included reviews, which was frequently incomplete.
• We did not assess the relevance of the reviews identified by the updated search or summarise the findings for any of those reviews.
• We did not consider publication bias in the included reviews. It is uncertain whether publication bias affected the results of the included reviews. To the extent it did, the results may overestimate the effects of the interventions.

### Implications

A coordinated effort by educational researchers and education professionals is needed to:

• Reach a consensus on terminology and definitions of teaching strategies,
• Set priorities for systematic reviews of teaching strategies for which a high quality, up-to-date systematic review cannot be found, and
• Reduce unnecessary duplication of efforts.

Investigators preparing prioritised systematic reviews should consider the limitations that we identified in the included reviews when preparing a protocol. They should consider potential adverse effects, systematically assess the risk of bias for included studies, and systematically assess the credibility of subgroup effects (effect modifiers).

Teachers and developers of educational resources for teaching critical thinking in primary and secondary schools may want to consider using some of the teaching strategies that we have judged to be most relevant. There are important uncertainties about the effects of most of those teaching strategies, and there may be other teaching strategies worth considering for which we did not find a systematic review. Nonetheless, the teaching strategies summarised provide a useful starting point for identifying and selecting potentially useful teaching strategies. ^73^

## Conclusions

A tremendous amount of work has gone into evaluating the effects of a wide range of teaching strategies. The results of this research can inform decisions about how to teach critical thinking or achieve other learning goals, as well as decisions about further research to evaluate the effects of teaching strategies. However, well-designed, up-to-date systematic reviews are still needed for many teaching strategies. Coordination of efforts could help to address this need.

### Ethics and consent

Ethics and consent were not required.

## Data Availability

Zenodo: Dataset for “The effects of teaching strategies on learning to think critically in primary and secondary schools: an overview of systematic reviews”, https://doi.org/10.5281/zenodo.14001300. ^73^ This project contains the following underlying data:
- Included systematic reviews.xlsx - Overview of teaching strategies.xlsx Zenodo: Dataset for “The effects of teaching strategies on learning to think critically in primary and secondary schools: an overview of systematic reviews”, https://doi.org/10.5281/zenodo.14001300. ^73^ This project contains the following extended data:
- Education Research Complete search strategy.docx - ERIC search strategy.docx -
PRISMA_2020_checklist teaching strategies.docx - Strategies for teaching critical thinking.docx Data are available under the terms of the Creative Commons Attribution 4.0 International license (CC-BY 4.0).
