# Supplementary material for: The effects of teaching strategies on learning to think critically in primary and secondary schools: an overview of systematic reviews
Source: F1000Res. 2025 Apr 16;13:1426. Originally published 2024 Nov 26. [Version 2] doi: 10.12688/f1000research.158087.2 (PMC12022541; doi:10.12688/f1000research.158087.2)
Supplement: Peer review history

# Reviewer report: Reviewer response for version 2

**DOI:** 10.5256/f1000research.180475.r378503

**Published:** 2025-04-24

**Authors:** Keji Fan

**Affiliations:** 1 Durham University, Durham, England, UK

The new version has addressed my concerns to a satisfactory level.

Are the rationale for, and objectives of, the Systematic Review clearly stated?

Yes

Is the statistical analysis and its interpretation appropriate?

Yes

If this is a Living Systematic Review, is the ‘living’ method appropriate and is the search schedule clearly defined and justified? (‘Living Systematic Review’ or a variation of this term should be included in the title.)

Yes

Are sufficient details of the methods and analysis provided to allow replication by others?

Yes

Are the conclusions drawn adequately supported by the results presented in the review?

Yes

Reviewer Expertise:

NA

I confirm that I have read this submission and believe that I have an appropriate level of expertise to confirm that it is of an acceptable scientific standard.

# Reviewer report: Reviewer response for version 1

**DOI:** 10.5256/f1000research.173632.r360693

**Published:** 2025-02-22

**Authors:** Keji Fan

**Affiliations:** 1 Durham University, Durham, England, UK

This overview provides a timely and important examination of current evidence synthesis in the field of critical thinking instruction. It points out issues such as the lack of consideration of adverse effects and the risks of bias assessment. However, several areas need to be addressed to enhance the overall quality:

 Introduction

 1. This section clearly defines key terms, such as critical thinking and teaching strategies. However, the justification for providing an overview of systematic reviews (SR) needs further clarification. Although the authors suggest that the overview will inform the design of their intervention, *Be Smart about Your Health*, it would strengthen the argument if they justified the necessity of the overview from both a methodological and a content-specific perspective regarding critical thinking instruction.

 Methods

 2. One of the objectives of this overview is to "inform the development of a framework for types of teaching strategies." However, pre-specifying teaching strategies could present potential issues. For instance, what if the included SRs identify additional teaching strategies? How can you check that the teaching strategies considered are comprehensive? What criteria were used to develop the list of teaching strategies, particularly given that some may overlap?

 3. Does the overview account for publication bias, particularly with studies that remain unpublished? If not, this should be acknowledged in the limitations section. Additionally, it would be useful to discuss whether and how the inclusion of grey literature could alter your findings.

 4. While you have used Doyle's framework on cognitive tasks, it is unclear how this framework relates to the development of your teaching strategies. Further explanation is needed. Additionally, some may argue that memorisation is not a critical thinking skill. How might this affect your study?

 5. I think it is a good idea to consider SRs in terms of risks of bias, sub-group effects and adverse effects. However, more justification is needed to explain why these aspects are important. In other words, why should we consider these factors? How might they influence the field of critical thinking instruction? A brief discussion on why previous reviews failed to consider adverse effects would also be beneficial. Is it possible that primary research included in these systematic reviews overlooked these factors?

 Results

 6. A summary paragraph outlining the specific aspects of the SRs you considered would be helpful. Why did you choose these aspects, such as countries, educational levels, and subject areas? It would also be useful to discuss how SRs conducted in these countries might inform the East African context. Are certain components transferable to your teaching environment? Furthermore, as educational levels differ across various educational systems, would it be biased to categorise studies solely by educational level?

 7. The categories in the tables may need rephrasing to make each one distinct. For example, in Table 3, the first category is "multiple subjects." How does this differ from other categories (I can see that there are explanations in the text)? Additionally, please check the consistency of the data between the text and the table. For instance, in the text, it states that "many of the reviews (112; 34.4%) included studies in multiple school subjects," whereas in the table, the number for multiple subjects is 113 (34.7%).

 Discussion

 8. While including countries and educational levels as categories to describe the current state of SR in critical thinking instruction is useful, it would be beneficial to discuss the possible reasons for these patterns. For example, why are critical thinking teaching reviews more or less prevalent in certain countries?

 9. The broad focus of the overview is well-justified; however, a discussion on how specific teaching strategies might differ or be transferable when teaching critical thinking in the context of health education would add value. Is critical thinking instruction in areas such as language, mathematics, or social-emotional learning the same as in health education?

 10. I agree with your justification that reported effect sizes should not be used to compare the effectiveness of teaching strategies due to their heterogeneity. This point could be made clearer in Figure 3, where average effect sizes from meta-analyses are presented. Could this be misleading if the average effect sizes are shown here?

Are the rationale for, and objectives of, the Systematic Review clearly stated?

Yes

Is the statistical analysis and its interpretation appropriate?

Yes

If this is a Living Systematic Review, is the ‘living’ method appropriate and is the search schedule clearly defined and justified? (‘Living Systematic Review’ or a variation of this term should be included in the title.)

Yes

Are sufficient details of the methods and analysis provided to allow replication by others?

Yes

Are the conclusions drawn adequately supported by the results presented in the review?

Yes

Reviewer Expertise:

Systematic reviews; evidence synthesis; critical thinking instruction; randomised controlled trials; evidence-based education

I confirm that I have read this submission and believe that I have an appropriate level of expertise to confirm that it is of an acceptable scientific standard, however I have significant reservations, as outlined above.

# Response

**Published:** 2025-04-09

**Authors:** Heather Munthe-Kaas

**Affiliations:** Norwegian Institute of Public Health, Norway

Introduction:

 1. This section clearly defines key terms, such as critical thinking and teaching strategies. However, the justification for providing an overview of systematic reviews (SR) needs further clarification. Although the authors suggest that the overview will inform the design of their intervention, *Be Smart about Your Health*, it would strengthen the argument if they justified the necessity of the overview from both a methodological and a content-specific perspective regarding critical thinking instruction.

**RESPONSE: **Thank you. We have added the following to the introduction to better situate this study in the broader context:

 “However, the results from this review can also be applied more broadly to inform the development of other critical thinking learning resources. Given the growing use of large language model and artificial intelligence that young people are exposed to, coupled with a deluge of mis/disinformation, critical thinking skills are increasingly important to ensure that young people have the necessary skills to identify reliable evidence and make informed choices. In response, many educational systems have begun to recognize the importance of critical thinking by including these skills in curriculum reforms, national and international standardized tests (e.g., Programme for International Student Assessment; PISA), and by incorporating problem-based learning approaches in the classroom.{Hafizah, 2024 #73} However, the implementation of teaching strategies to facilitate and support the development of critical thinking skills varies widely across subjects, schools and countries.{Johnston, 2023 #71}{Rott, 2024 #72} “

 Methods:

 2. One of the objectives of this overview is to "inform the development of a framework for types of teaching strategies." However, pre-specifying teaching strategies could present potential issues. For instance, what if the included SRs identify additional teaching strategies? How can you check that the teaching strategies considered are comprehensive? What criteria were used to develop the list of teaching strategies, particularly given that some may overlap?

**RESPONSE: **Thank you. We used a common taxonomy from Beck (that was based on a review of teacher education textbooks) to initially develop a list of potentially relevant teaching strategies. We further developed this list iteratively based on literature review and stakeholder engagement. The protocol provides a comprehensive overview of how the search strategy and methods were developed.

https://informedhealthchoices.org/wp-content/uploads/2022/12/Teaching-strategies-overview-protocol.pdf

 We agree that there is inherent uncertainty about whether our framework is useful and fully comprehensive, which is why we stated in the discussion: “…It would be helpful for users of this research if researchers studying the effects of teaching strategies could reach a consensus on a taxonomy.”

 It was beyond the scope of this review to report on additional teaching strategies that were not aiming to teach critical thinking.

 3. Does the overview account for publication bias, particularly with studies that remain unpublished? If not, this should be acknowledged in the limitations section. Additionally, it would be useful to discuss whether and how the inclusion of grey literature could alter your findings.

**RESPONSE: **Thank you. We have added the following limitation under the Discussion section

 “We did not consider publication bias in the included reviews. It is uncertain whether publication bias affected the results of the included reviews. To the extent it did, the results may overestimate the effects of the interventions.”

 ERIC and Education Research Complete include some grey literature. We did not otherwise conduct any specific searches for grey literature. The detailed search strategies are in Appendices A and B.

 4. While you have used Doyle's framework on cognitive tasks, it is unclear how this framework relates to the development of your teaching strategies. Further explanation is needed. Additionally, some may argue that memorisation is not a critical thinking skill. How might this affect your study?

**RESPONSE: **Thank you. Doyle’s framework was used to define or limit “teaching” to something that is intended to promote skills as described by Doyle (which corresponds roughly with Bloom’s taxonomy) as opposed to teaching that may seek to improve emotions or attitude (for example teaching aimed at improving well-being or interest in science). These skills were not limited to those that are ”critical thinking skills” but rather to help limit what “teaching” is in the context of this review.

 5. I think it is a good idea to consider SRs in terms of risks of bias, sub-group effects and adverse effects. However, more justification is needed to explain why these aspects are important. In other words, why should we consider these factors? How might they influence the field of critical thinking instruction? A brief discussion on why previous reviews failed to consider adverse effects would also be beneficial. Is it possible that primary research included in these systematic reviews overlooked these factors?

 I am not sure I understand this comment. It is common practice to extract these from SRs in a systematic review of reviews (OoO). We do not contend that previous SRs have failed to consider these factors, but rather are adhering to best practice in a systematic review of reviews. Risk of bias is unlikely to have been assessed by the primary study, subgroup effects may also not be reported in all studies, but may have only been possible to calculate in a SR. It is not always clear whether primary studies report adverse effects, and any discussion of why previous reviews failed to consider adverse effects would be speculation, as we have no clues as to why. 

 Results

 6. A summary paragraph outlining the specific aspects of the SRs you considered would be helpful. Why did you choose these aspects, such as countries, educational levels, and subject areas? It would also be useful to discuss how SRs conducted in these countries might inform the East African context. Are certain components transferable to your teaching environment? Furthermore, as educational levels differ across various educational systems, would it be biased to categorise studies solely by educational level?

**RESPONSE: **We have one paragraph each describing the country, educational level and subject areas of the primary studies included in the included SRs (see paragraphs above Tables 1, 2 and 3). Because educational levels differ, we categorized broadly using terms such as “preschool”, “primary school” etc. While we acknowledge that the exact years included in each of these types of school may differ, this was the best effort at presenting categorization. We did not categorize solely by educational level, but also by school subjects.

 We discuss transferability in the discussion section “Nonetheless, the findings of the systematic reviews that we considered most relevant for teaching critical thinking in primary and secondary schools helped to inform the design of the Be Smart about your Health resources. Because we developed the resources in East Africa, many of the teaching strategies that we considered potentially relevant in other settings were not practical in low-resource settings, particularly ones that depend on the availability of digital technology.”

 7. The categories in the tables may need rephrasing to make each one distinct. For example, in Table 3, the first category is "multiple subjects." How does this differ from other categories (I can see that there are explanations in the text)? Additionally, please check the consistency of the data between the text and the table. For instance, in the text, it states that "many of the reviews (112; 34.4%) included studies in multiple school subjects," whereas in the table, the number for multiple subjects is 113 (34.7%).

**RESPONSE: **Thank you. There is a challenge in naming the categories because they are not always distinct and the limitation of the table format. We hope the text supports the reader in understanding the results.

 Thank you – we have now corrected so that n=112 in the table and the text.

 Discussion:

 8. While including countries and educational levels as categories to describe the current state of SR in critical thinking instruction is useful, it would be beneficial to discuss the possible reasons for these patterns. For example, why are critical thinking teaching reviews more or less prevalent in certain countries?

**RESPONSE: **Thank you. We believe this is very interesting, but is beyond the scope of this review and would require either speculation or a deep understanding of multiple countries and their sociocultural perspectives and values connected to critical thinking, media literacy, health literacy and/or trust in government for example

 9. The broad focus of the overview is well-justified; however, a discussion on how specific teaching strategies might differ or be transferable when teaching critical thinking in the context of health education would add value. Is critical thinking instruction in areas such as language, mathematics, or social-emotional learning the same as in health education?

**RESPONSE: **Thank you. This is very interesting, but outside the scope of this review and under researched in the literature. We have added the following to address this point “Some of the teaching strategies included in this review may be more effective for some subjects than others, however this is outside the scope of this review, but could benefit from further investigation.“

 10. I agree with your justification that reported effect sizes should not be used to compare the effectiveness of teaching strategies due to their heterogeneity. This point could be made clearer in Figure 3, where average effect sizes from meta-analyses are presented. Could this be misleading if the average effect sizes are shown here?

**RESPONSE:** Suggestion to change the Figure label to “Average effect sizes from meta-analyses included in summaries of the most relevant teaching strategies **vary greatly”**

# Reviewer report: Reviewer response for version 1

**DOI:** 10.5256/f1000research.173632.r345795

**Published:** 2025-01-28

**Authors:** Sofia Avgitidou

**Affiliations:** 1 Aristotle University of Thessaloniki, Thessaloniki, Greece

This an interesting paper with clear objectives and criteria for a systematic review of other reviews on teaching strategies and their effects on learning and critical thinking in education. Despite the lack of some evidence in the reviews that were anaysed, the study contributes to the categorisation of teaching strategies and in summarizing their (possible) effects on learning.

Are the rationale for, and objectives of, the Systematic Review clearly stated?

Yes

Is the statistical analysis and its interpretation appropriate?

Yes

If this is a Living Systematic Review, is the ‘living’ method appropriate and is the search schedule clearly defined and justified? (‘Living Systematic Review’ or a variation of this term should be included in the title.)

Not applicable

Are sufficient details of the methods and analysis provided to allow replication by others?

Yes

Are the conclusions drawn adequately supported by the results presented in the review?

Yes

Reviewer Expertise:

Initial and in-service teacher education, professional learning, transformation of education and educational change, democratic and participatory education

I confirm that I have read this submission and believe that I have an appropriate level of expertise to confirm that it is of an acceptable scientific standard.

# Response

**Published:** 2025-04-09

**Authors:** Heather Munthe-Kaas

**Affiliations:** Norwegian Institute of Public Health, Norway

Thank you for your comment.
